# Intravenous Immunoglobulin Use in Hemolytic Disease Due to ABO Incompatibility to Prevent Exchange Transfusion

**DOI:** 10.3389/fped.2022.864609

**Published:** 2022-04-28

**Authors:** Emel Okulu, Omer Erdeve, Ilknur Kilic, Ozgur Olukman, Sebnem Calkavur, Gokhan Buyukkale, Merih Cetinkaya, Dilek Ulubas, Nihal Demirel, Deniz Hanta, Sabahattin Ertugrul, Nazli Dilay Gultekin, Oguz Tuncer, Nihat Demir, Leyla Bilgin, Nejat Narli, Duran Yildiz, Demet Terek, Ozge Altun Koroglu, Canan Seren, Elif Ozyazici, Ramazan Ozdemir, Hatice Turgut, Fatma Narter, Yasemin Akin, Ahmet Ozyazici, Aysegul Zenciroglu, Huseyin Selim Asker, Zeynel Gokmen, Musa Salihli, Ali Bulbul, Umut Zubarioglu, Begum Atasay, Esin Koc, Turkish Neonatal Society IVIG Study Group, Kurthan Mert

**Affiliations:** ^1^Division of Neonatology, Department of Pediatrics, Faculty of Medicine, Ankara University, Ankara, Turkey; ^2^Department of Neonatology, Atasehir Florence Nightingale Hospital, Istanbul, Turkey; ^3^Department of Neonatology, Izmir Behcet Uz Children's Hospital, University of Health Sciences, Izmir, Turkey; ^4^Department of Neonatology, Kanuni Sultan Suleyman Training and Research Hospital, University of Health Sciences, Istanbul, Turkey; ^5^Department of Neonatology, Etlik Zubeyde Hanim Women's Health Teaching and Research Hospital, University of Health Sciences, Ankara, Turkey; ^6^Division of Neonatology, Department of Pediatrics, Faculty of Medicine, Yildirim Beyazit University, Ankara, Turkey; ^7^Department of Neonatology, Adana Delivery and Child Disease Hospital, Adana, Turkey; ^8^Division of Neonatology, Department of Pediatrics, Faculty of Medicine, Dicle University, Diyarbakir, Turkey; ^9^Division of Neonatology, Department of Pediatrics, Meram Faculty of Medicine, Necmettin Erbakan University, Konya, Turkey; ^10^Division of Neonatology, Department of Pediatrics, Faculty of Medicine, Yuzuncuyil University, Van, Turkey; ^11^Department of Neonatology, Umraniye Research and Training Hospital, University of Health Sciences, Istanbul, Turkey; ^12^Neonatal Intensive Care Unit, Adana Metro Hospital, Adana, Turkey; ^13^Nenehatun Obstetrics and Gynecology Hospital, Erzurum, Turkey; ^14^Division of Neonatology, Department of Pediatrics, Faculty of Medicine, Ege University, Izmir, Turkey; ^15^Division of Neonatology, Department of Pediatrics, Faculty of Medicine, Ondokuz Mayis University, Samsun, Turkey; ^16^Division of Neonatology, Department of Pediatrics, Faculty of Medicine, Inonu University, Malatya, Turkey; ^17^Department of Neonatology, Kartal Dr. Lutfi Kirdar Training and Research Hospital, University of Health Sciences, Istanbul, Turkey; ^18^Department of Neonatology, Dr. Sami Ulus Research and Training Hospital of Women's and Children's Health and Diseases, Ankara, Turkey; ^19^Neonatal Intensive Care Unit, NCR Hospital, Gaziantep, Turkey; ^20^Division of Neonatology, Department of Pediatrics, Faculty of Medicine, Konya Hospital, Başkent University, Konya, Turkey; ^21^Sisli Hamidiye Etfal Training and Research Hospital, University of Health Sciences, Istanbul, Turkey; ^22^Division of Neonatology, Department of Pediatrics, Faculty of Medicine, Gazi University, Ankara, Turkey

**Keywords:** hemolytic disease of the newborn, ABO incompatibility, intravenous immunoglobulin, exchange transfusion, light-emitting diode, phototherapy

## Abstract

**Introduction:**

Intravenous immunoglobulin (IVIG) has been widely used to treat the hemolytic disease of the newborn (HDN). Although it has been shown that IVIG treatment reduces the duration of phototherapy and hospitalization, the use of IVIG in hemolytic disease due to ABO incompatibility has been controversial in recent years. This study aimed to investigate the role of IVIG in the prevention of exchange transfusion in infants with ABO HDN who presented with bilirubin levels at or above the level of exchange transfusion.

**Materials and Methods:**

This study evaluated the data of infants with ABO HDN in the Turkish Neonatal Jaundice Online Registry. The infants with ABO HDN who met the total serum bilirubin level inclusion criteria (within 2–3 mg/dL of exchange transfusion or even above exchange transfusion level) were included in the study according to the guidelines from the American Academy of Pediatrics and the Turkish Neonatal Society. All patients were managed according to the unit protocols recommended by these guidelines and received light-emitting diode (LED) phototherapy. Infants who only received LED phototherapy, and who received one dose of IVIG with LED phototherapy were compared.

**Results:**

During the study period, 531 term infants were included in the study according to inclusion criteria. There were 408 cases in the phototherapy-only group, and 123 cases in the IVIG group. The demographic findings and the mean bilirubin and reticulocyte levels at admission were similar between the groups (*p* > 0.05), whereas the mean hemoglobin level was slightly lower in the IVIG group (*p* = 0.037). The mean age at admission was earlier, the need for exchange transfusion was higher, and the duration of phototherapy was longer in the IVIG group (*p* < 0.001, *p* = 0.001, and *p* < 0.001, respectively). The rate of re-hospitalization and acute bilirubin encephalopathy (ABE) was higher in the IVIG group (*p* < 0.001 and *p* = 0.01, respectively).

**Conclusion:**

In this study, we determined that one dose of IVIG did not prevent an exchange transfusion nor decrease the duration of phototherapy in infants, who had bilirubin levels near or at exchange transfusion level, with hemolytic disease due to ABO incompatibility.

## Introduction

Hemolytic disease of the newborn (HDN) that is due to the destruction of antibody-coated red cells through the reticuloendothelial system and complement activation may cause severe hyperbilirubinemia. ABO HDN has been reported to be the most common alloimmune hemolytic disease in neonates. ABO incompatibility affects about 25% of all maternal/fetal couples, while ABO HDN affects <1% of the group of women with antenatal high titer immunoglobulin (IgG) antibodies (1).

Phototherapy for neonatal jaundice is the most common therapeutic modality in neonatal intensive care units (NICUs). Exchange transfusion is an effective treatment option in severe cases but is also an invasive procedure with significant risks (2, 3). In recent years, intravenous immunoglobulin (IVIG) has been used to treat infants with ABO HDN. It has been shown that IVIG treatment reduces the duration of phototherapy and hospitalization, and the need for exchange transfusion, but there is still scant information on this subject. A recent Cochrane review found insufficient evidence to recommend the use of IVIG in alloimmune HDN (4). IVIG is derived from pooled human plasma, so there is a risk of significant morbidities such as necrotizing enterocolitis and severe hemolysis in neonates (5, 6).

The Turkish Neonatal Society established the Turkish Neonatal Jaundice Online Registry to estimate the incidence of severe neonatal jaundice and facilitate a root cause analysis of neonatal jaundice and its complications. This nationwide database showed that hemolytic jaundice was the leading cause of neonatal jaundice, in which ABO incompatibility is the most common (7). Therefore, we performed a secondary analysis of this observational multicenter Turkish Neonatal Jaundice Online Registry database to investigate the role of IVIG in the prevention of exchange transfusion in ABO HDN.

## Materials and Methods

This study utilized the data of infants with ABO HDN in the Turkish Neonatal Jaundice Online Registry. The infants with ABO HDN, who met the total serum bilirubin level inclusion criteria (within 2–3 mg/dL of exchange transfusion or even above exchange transfusion level), were included in the study according to the guidelines from the American Academy of Pediatrics and the Turkish Neonatal Society (8, 9). All patients were managed according to the unit protocols recommended by these guidelines and received light-emitting diode (LED) phototherapy.

The hemolytic disease was defined by anemia, reticulocytosis, peripheral smear findings, positive direct antiglobulin test, and/or positive antibody on infant's red blood cells. One dose of IVIG is recommended to administer (0.5–1 g/kg over 2 h) for the treatment of isoimmune hemolytic disease when the total serum levels of bilirubin increase despite intensive phototherapy or if levels are 2–3 mg/dL of the exchange level (8, 9). Each unit gave its own decision to use or not to use IVIG for the treatment. Exchange transfusion was also performed as needed (8, 9). The bilirubin-induced neurological dysfunction score was used to identify acute bilirubin encephalopathy (10).

Infants who filled the inclusion criteria were classified into two groups: (1) the phototherapy group that received only LED phototherapy and (2) the IVIG group that received one dose of IVIG plus LED phototherapy. Data on patient demographics, as well as clinical and laboratory findings, duration of phototherapy, need for exchange transfusion, and the incidence of bilirubin-induced neurological sequelae were evaluated regarding the treatment for jaundice.

Online data were summarized using descriptive statistics. Categorical data are presented as numbers (n) and percentages (%). Continuous variables are presented as mean ± standard deviation. The chi-square or Fisher exact test was used for statistical comparisons. A *p-value* of 0.05 level was considered statistically significant.

## Results

**Figure 1:**
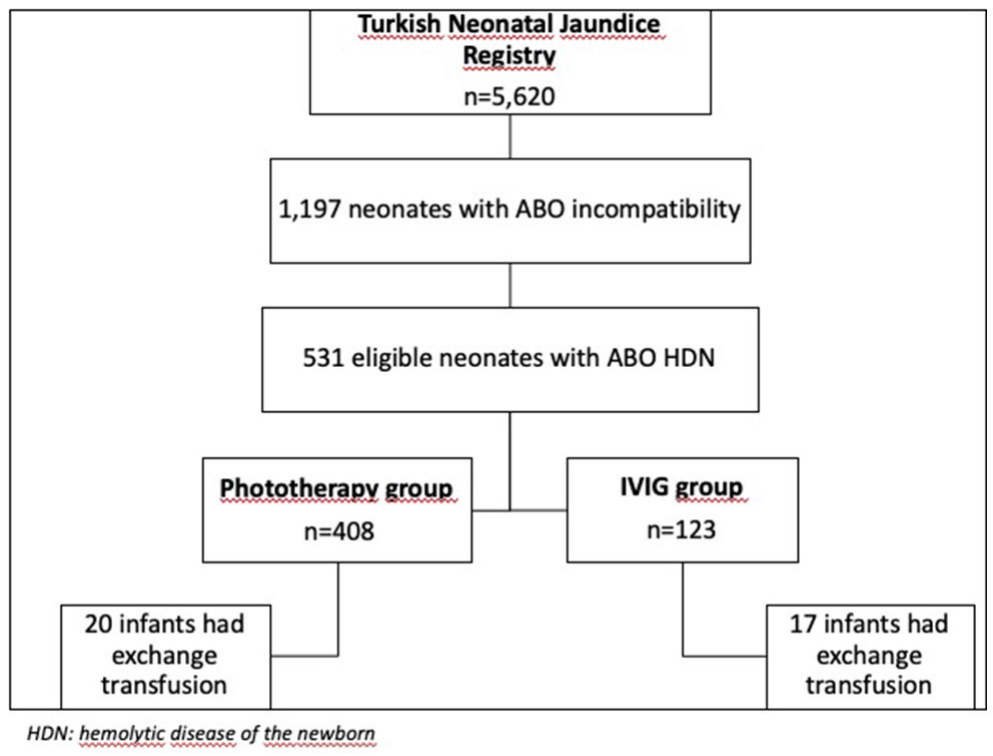
The flowchart of the study.

During the study period, 1,197 infants were recorded as ABO incompatibility in the “Turkish Neonatal Jaundice Online Registry,” and 531 of them were included in the study according to inclusion criteria as ABO HDN. There were 408 cases in the phototherapy-only group, and 123 cases in the IVIG group (Figure 1).

The demographic and clinical characteristics of the study cohort are summarized in Table 1. There were no significant differences between the two groups concerning gestational age, birth weight, gender or bilirubin level, and reticulocyte count at admission (*p* > 0.05). The mean postnatal age at admission and hemoglobin level were higher in the phototherapy group (*p* < 0.001 and 0.037, respectively), whereas the mean duration of phototherapy was longer in the IVIG group (*p* < 0.001).

**Table 1:** Demographic and clinical characteristics of the infants.

	**Phototherapy group (*n* = 408)**	**IVIG group (*n* = 123)**	***p***
Gestational age (weeks)^*^	38.4 ± 1.3	38.6 ±1.3	0.053
Birth weight (g)^*^	3,200 ± 435	3,206 ± 434	0.89
Gender (female), *n* (%)	194 (46)	63 (51)	0.53
Postnatal age at admission (d)^*^	4.1 ± 1.7	2.9 ± 1.7	<0.001
Bilirubin level (mg/dL)^*^	21.1 ± 3.8	21.6 ± 5.6	0.239
Hemoglobin level (g/dL)^*^	15.2 ± 2	14.8 ± 2.1	0.037
Reticulocyte count (%)^*^	2.7 ± 1.7	2.7 ± 2	0.95
Exchange transfusion, *n* (%)	20 (5)	17 (14)	0.001
Duration of phototherapy (h)^*^	35.6 ±19.2	50 ±25.8	<0.001

* *Data given as mean ± SD*.

**Figure 2:**
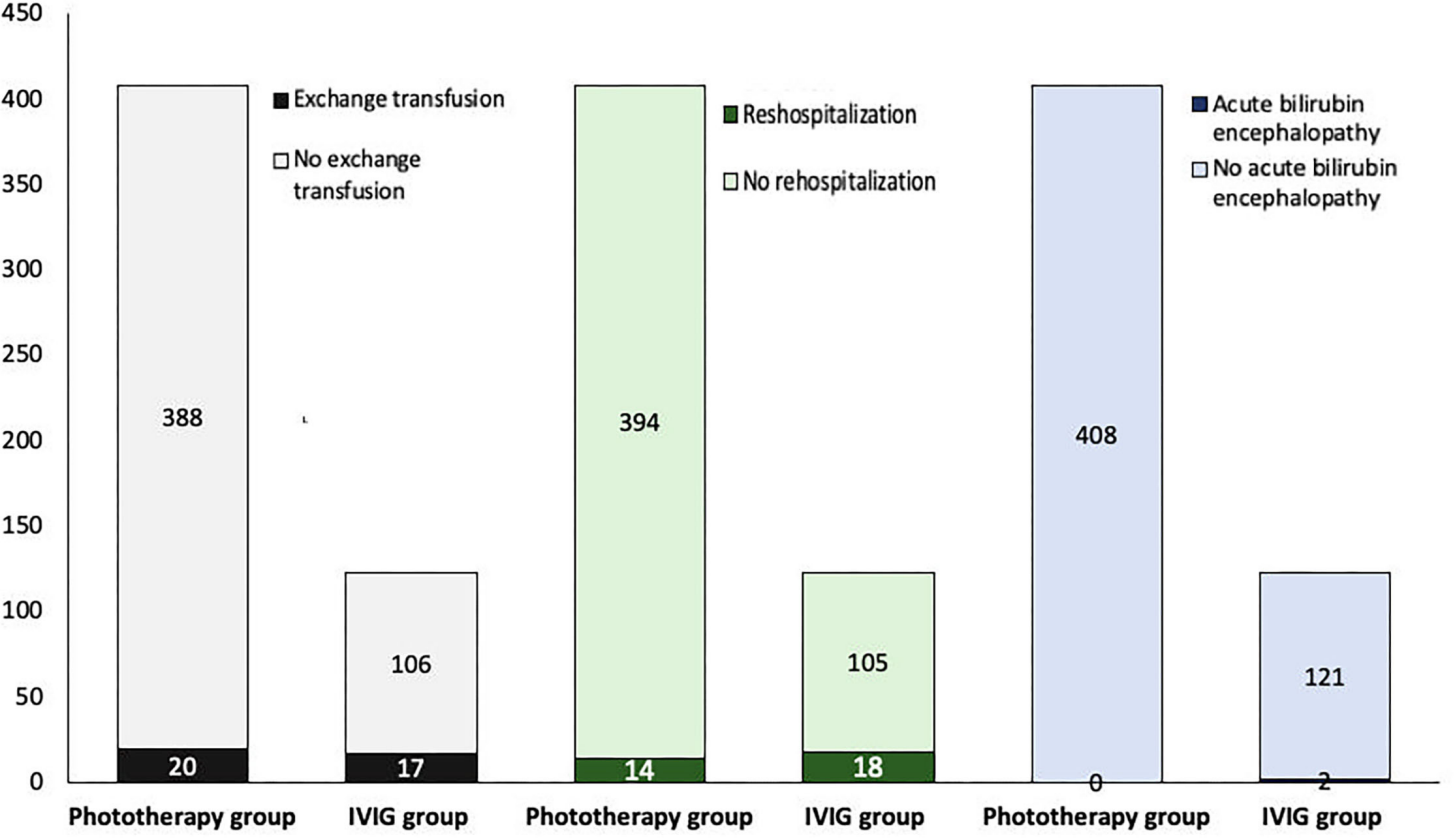
Intravenous immunoglobulin (IVIG) has no advantage in avoiding exchange transfusion, rehospitalization, and neurological sequelae (*p* = 0.001, <0.001, and 0.01, respectively).

Twenty infants (5%) in the phototherapy group and 17 infants (14%) in the IVIG group received an exchange transfusion (Table 1). There was a significant difference between groups concerning exchange transfusion, which was higher in infants who received IVIG as an adjunct treatment to phototherapy (*p* = 0.001) (Figure 2). The included infants in the IVIG group received IVIG at a single dose of 0.5–1 g/kg.

Complications related to exchange transfusion were observed in 6 (16%) of 37 infants who underwent exchange transfusion. There were four cases of thrombocytopenia, and two of hypocalcemia. No adverse effect associated with IVIG was observed in any patients.

The rate of rehospitalization and acute bilirubin encephalopathy (ABE) was higher in the IVIG group (*p* < 0.001, and *p* = 0.01, respectively) (Table 2). ABE occurred in two infants in the IVIG group who also underwent exchange transfusion (Figure 2).

**Table 2:** Outcomes of infants.

	**Phototherapy group (*n* = 408)**	**IVIG group (*n* = 123)**	***p***
Rehospitalization, *n* (%)	14 (3.4)	18 (14.6)	<0.001
Hearing impairment, *n* (%)	1 (0.2)	2 (1.6)	0.073
Acute bilirubin encephalopathy, *n* (%)	0 (0)	2 (1.6)	0.01

## Discussion

The use of IVIG was first shown to be effective for treating blood group incompatibility in the 1990's (11). Since then, it has been used to treat HDN to avoid exchange transfusion, but there is still no consensus on its use. In this study, we found that one dose of IVIG in infants who were admitted with ABO HDN at a bilirubin level within 2–3 mg/dL of the exchange transfusion level or even above the exchange transfusion level did not prevent an exchange transfusion.

IVIG is derived from the pooled human plasma, which means it is obtained from several thousand healthy donors, so there is a potential risk of transfusion-transmitted disease. The mechanism of IVIG is unclear, but it is thought that IVIG decreases hemolysis by blocking Fc receptors in the reticuloendothelial system and preventing the lysis of antibody-coated erythrocytes (12, 13). Although several studies have suggested that IVIG was effective for treating neonatal hemolytic jaundice and decreasing the number of infants that require an exchange transfusion (14–18), some clinical studies found that IVIG treatment failed in the management of infants with ABO HDN (19, 20). Recently, a Cochrane review found insufficient evidence to recommend the use of IVIG in alloimmune HDN (4).

The need for exchange transfusion treatment after IVIG use has varied among studies. Many factors, including age upon admission to the hospital, duration and strength of phototherapy, and dose and time of administration of IVIG may influence the response to IVIG (11, 17, 21). Hammerman et al. reported that infants who did not respond sufficiently to IVIG therapy have higher carboxy-hemoglobin levels, which means that their hemolysis was more severe (22). In this study, infants who received IVIG were admitted to the hospital earlier with similar bilirubin (mg/dL) and reticulocyte (%) levels, and slightly lower hemoglobin (g/dL) levels compared to the phototherapy group. However, the need for exchange transfusion was higher, and the duration of phototherapy was longer in the IVIG group. The efficacy of phototherapy is dependent on the wavelength and intensity of the light emitted during phototherapy, the duration, and surface area of the body exposure (23). The American Academy of Pediatrics defines intensive phototherapy as irradiance of at least 30 mW/(cm^2^ nm) in the 430–490 nm band (8). High-intensity LEDs have been developed for intensive phototherapy. All infants included in this study received LED phototherapy. It is suggested that the recent advanced phototherapy method is effective to reduce the bilirubin levels, and the addition of IVIG did not decrease the need for an exchange transfusion.

Acute and chronic bilirubin encephalopathy is largely preventable if severe hyperbilirubinemia is identified early and treated promptly. Despite the availability of effective treatment, severe hyperbilirubinemia and its consequences are seen in a wide range of occurrences around the world, depending on the control measures used (24–26). The clinical risk factors associated with neurotoxicity at lower serum bilirubin levels include hemolytic disease, glucose-6-phosphate dehydrogenase deficiency, prematurity, asphyxia, acidosis, sepsis, and hypoalbuminemia (27–29). This study included infants with HDN due to ABO incompatibility, who are known to be at risk for neurological dysfunction.

Our study has some strengths and limitations. Data of infants who have ABO HDN were taken from the Turkish Neonatal Jaundice Registry, which consists of self-reported data. This study is a retrospective secondary analysis of the observational data; therefore, it was not powered to analyze the impact of IVIG in the prevention of exchange transfusion as a primary outcome. Since one dose of IVIG was administrated to infants with bilirubin levels near the exchange transfusion level, the effect of IVIG might be greater when the bilirubin level was not as high, and it is uncertain if two doses of IVIG would have been effective. However, this study enrolled the largest number of ABO HDN term infants, who received one dose of IVIG plus LED phototherapy vs. LED phototherapy alone. Second, all cases were reported from tertiary NICUs, where neonatologists strictly follow bilirubin treatment guidelines.

## Conclusion

Some NICUs administer IVIG to term infants with ABO HDN with a bilirubin level at or above the exchange transfusion level. In this observational study, we demonstrated that one dose of IVIG treatment did not reduce or prevent the need for exchange transfusion nor did it decrease the duration of phototherapy in this population. The efficacy of advanced technology in phototherapy may decrease the effectiveness of IVIG to prevent exchange transfusion in this group. Additional clinical studies are needed to determine the appropriate use of IVIG in the treatment of ABO HDN.

## Data Availability Statement

The raw data supporting the conclusions of this article will be made available by the authors, without undue reservation.

## Ethics Statement

The studies involving human participants were reviewed and approved by Ankara University Institutional Review Board (Approval No. 14-593-15). Written informed consent to participate in this study was provided by the participants' legal guardian/next of kin.

## Author Contributions

EOk and OE gave a substantial contribution to article conception and design. IK, OO, SC, GB, MC, DU, NihalD, DH, SE, NG, OT, NihatD, LB, NN, DY, DT, OK, CS, EOz, RO, HT, FN, YA, AO, AZ, HA, ZG, MS, AB, UZ, and EK participated in acquisition of data. EOk and OE drafted the manuscript. BA critically revised it. All authors gave their final approval to this manuscript and agree to be accountable for all aspects of the work ensuring integrity and accuracy.

## Funding

This study was supported by the Turkish Neonatal Society, and the financial fund was used to create the Turkish Neonatal Jaundice Online Registry database.

## Turkish Neonatal Society Ivig Study Group

We thank to Turkish Neonatal Society for supporting the project, all collaborator NICUs and the following investigators, as the members of the Turkish Neonatal Society IVIG Study Group: Kurthan Mert (Numune Education and Research Hospital, Adana, Turkey); Akan Yaman (Sanliurfa Maternity Hospital, Sanliurfa, Turkey); Evrim Alyamac Dizdar, Nurdan Uras (Zekai Tahir Burak Women's Health and Research Hospital, Ankara, Turkey); Berna Hekimoglu (Kanuni Education and Research Hospital, Trabzon, Turkey); Ayse Engin Arisoy, Yasemin Senel (Kocaeli University Faculty of Medicine, Kocaeli, Turkey); Eren Ozek, Hulya Ozdemir (Marmara University Faculty of Medicine, Istanbul, Turkey); Kadir Tekgunduz, Ibrahim Caner (Ataturk University Faculty of Medicine, Erzurum, Turkey); Sema Tanriverdi (Tokat State Hospital, Tokat, Turkey); Dilek Sarici, F. Mehmet Kislali (Kecioren Education and Research Hospital, Ankara, Turkey); Didem Aliefendioglu, Nilufer Guzoglu (Kirikkale University Faculty of Medicine, Kirikkale Turkey); Cumhur Aydemir (Bulent Ecevit University Faculty of Medicine, Zonguldak, Turkey); Muhittin Celik (Diyarbakir Children's Hospital, Diyarbakir, Turkey); Belma Saygili Karagol (Yenimahalle Education and Research Hospital, Ankara, Turkey); Bilge Bayraktar (Bezmialem University Faculty of Medicine, Istanbul, Turkey); Suzan Sahin (Adnan Menderes University Faculty of Medicine, Aydin, Turkey); Ibrahim Murat Hirfanoglu (Gazi University Faculty of Medicine, Ankara, Turkey); Betul Acunas (Trakya University Faculty of Medicine, Edirne, Turkey); Ferda Ozlu (Cukurova University Faculty of Medicine, Adana, Turkey); Ayse Ecevit (Başkent University Faculty of Medicine, Ankara Hospital, Ankara, Turkey); Cengiz Arcagok (Mus State Hospital, Mus, Turkey); Neslihan Tekin (Osmangazi University Faculty of Medicine, Eskisehir, Turkey); Hacer Ergin (Pamukkale University Faculty of Medicine, Denizli, Turkey); Selda Arslan (Mustafa Kemal University Faculty of Medicine, Hatay, Turkey); and Asuman Coban (Istanbul University Faculty of Medicine, Istanbul, Turkey).

## Conflict of Interest

The authors declare that the research was conducted in the absence of any commercial or financial relationships that could be construed as a potential conflict of interest.

## Publisher's Note

All claims expressed in this article are solely those of the authors and do not necessarily represent those of their affiliated organizations, or those of the publisher, the editors and the reviewers. Any product that may be evaluated in this article, or claim that may be made by its manufacturer, is not guaranteed or endorsed by the publisher.
